# GADD34 activates p53 and may have utility as a marker of atherosclerosis

**DOI:** 10.3389/fmed.2023.1128921

**Published:** 2023-05-09

**Authors:** Go Tomiyoshi, Rika Nakamura, Natsuko Shinmen, Yoichi Yoshida, Seiichiro Mine, Toshio Machida, Katsuro Iwase, Yasuo Iwadate, Takaki Hiwasa, Hideyuki Kuroda

**Affiliations:** ^1^Medical Project Division, Research Development Center, Fujikura Kasei Co., Saitama, Japan; ^2^Department of Biochemistry and Genetics, Graduate School of Medicine, Chiba University, Chiba, Japan; ^3^Department of Neurological Surgery, Graduate School of Medicine, Chiba University, Chiba, Japan; ^4^Department of Neurological Surgery, Chiba Prefectural Sawara Hospital, Chiba, Japan; ^5^Department of Neurosurgery, Chiba Cerebral and Cardiovascular Center, Chiba, Japan; ^6^Department of Neurosurgery, Eastern Chiba Medical Center, Chiba, Japan

**Keywords:** GADD34, ischemic stroke, atherosclerosis, p53, ubiquitination

## Abstract

We previously identified growth arrest and DNA-damage-inducible gene 34 (GADD34) as a marker of ischemic stroke. In the present study, serum levels of anti-GADD34 antibodies were found to be significantly higher in patients with acute ischemic stroke or chronic kidney disease compared to healthy donors. We then examined the biological function of GADD34 by transfection into U2OS human osteosarcoma and U87 human glioblastoma cells. Knockdown of GADD34 by siRNA resulted in enhanced cell proliferation, which was reversed by co-knockdown of MDM2. Luciferase reporter assays revealed that the transactivation ability of p53 enhanced by genotoxic anticancer drugs such as camptothecin and etoposide was further potentiated by enforced expression of GADD34 but attenuated by co-transfection with p53 shRNA expression plasmids. Western blotting demonstrated increased p53 protein levels after treatment with camptothecin, which was also potentiated by GADD34 but suppressed by GADD34 siRNA, ATM siRNA, and ATM inhibitor wortmannin. GADD34 levels also increased in response to treatment with camptothecin or adriamycin, and this increase was attenuated by MDM2 siRNA. Immunoprecipitation with anti-GADD34 antibody followed by Western blotting with anti-MDM2 antibodies indicated ubiquitination of GADD34 is mediated by MDM2. Accordingly, GADD34 may function as a ubiquitination decoy to reduce p53 ubiquitination and increase p53 protein levels. Increased neuronal cell death due to activation of p53 by GADD34 may account for the elevated serum levels of anti-GADD34 antibodies observed in patients with acute ischemic stroke.

## 1. Introduction

Mutations in the *p53* gene are the most frequently observed mutations in many cancer types (1). The major function of p53 is the suppression of tumorigenesis, with deletions or mutations in p53 frequently observed during oncogenesis (2, 3). p53 consists of an N-terminal transactivation domain, a DNA-binding domain, and a tetramerization domain. Wild-type p53 has a range of functions including as a transcription factor and the regulation of the cell cycle, DNA repair, and apoptosis. p53 can induce cell cycle arrest by transactivating p21, GADD45, and 14–3-3 (4) and induce apoptosis by direct binding to Bcl-2 or transactivation of Bax, Noxa, and Puma (5–7). The transactivation ability of p53 can be examined by luciferase reporter assays using reporter plasmids containing the promoter regions of p53-target genes conjugated to the luciferase gene. Thus, many reporter plasmids for p53 have been developed such as pG13-Luc (4), PUMA-Luc (7), p21-Luc (4), pGL3-Bax-Luc (5), Noxa-Luc (6), and Decorin-Luc (8), with pG13-Luc considered the most sensitive as the luciferase reporter contains 13 repeats of the consensus sequence of the p53-binding sequence.

p53 is known to be phosphorylated by ATM, ATR, and DNA-PK in response to DNA damage, resulting in decreased degradation and increased protein levels of p53 (9). MDM2, a p53-target gene, can ubiquitinate p53 leading to p53 degradation (10, 11). As the transactivation ability of p53 is almost entirely dependent on p53 protein levels, ubiquitination of p53 by MDM2 can modulate the activity of p53 as a transcription factor.

We previously identified growth arrest and DNA-damage-inducible gene 34 (GADD34; protein phosphatase 1, regulatory subunit 15A/PPP1R15A) as an antigen recognized by serum IgG antibodies in patients with atherosclerosis and acute ischemic stroke (AIS) (12). Recently, GADD34 was reported to be associated with p53 phosphorylation in ischemic stroke (13). ATM, which is able to phosphorylate and activate p53, has been shown to be involved in neuronal ischemic preconditioning and lethal ischemic injury (14). Thus, the present study examined the effects of GADD34 on p53.

## 2. Materials and methods

### 2.1. Chemicals

Camptothecin (CPT) was purchased from Biomol Research Laboratories (Plymouth Meeting, PA). Etoposide (also known as VP-16) was purchased from Nippon Kayaku (Tokyo, Japan). Adriamycin (ADM), wortmannin, and MG132 were purchased from Merck (Darmstadt, Germany). Dimethyl sulfoxide (DMSO) was purchased from FUJIFILM Wako Pure Chemical Corporation (Osaka, Japan).

### 2.2. Patients and healthy donor sera

The protocols of the present study were in compliance with the 1975 Declaration of Helsinki and approved by the Local Ethical Review Board of the Chiba University, Graduate School of Medicine in Chiba, Japan (No. 2018-320, 2020-1129) and the review boards of Sawara Hospital. Sera were collected from patients after provision of written informed consent. Serum samples from healthy donors (HD) and patients with AIS or transient ischemic attack (TIA) were obtained from Sawara Hospital as described previously (15). The stroke subtypes were assessed using the Trial of Org 10172 in the Acute Stroke Treatment categorization system (16), and AIS or ischemic stroke included large-artery atherosclerosis or minor arterial occlusion (lacune). HD subjects were selected as those without prior cancer history, autoimmune disease, or cerebro- and cardiovascular disease and exhibiting no abnormalities in cranial MRI. The sera of patients with chronic kidney disease (CKD) were obtained from the Kumamoto cohort (17, 18), of which the subject information used in the correlation analysis (Table 1) was summarized in Supplementary Table S1. Each serum sample was centrifuged at 3,000*g* for 10 min and supernatant was stored at −80°C until use. Repeated thawing and freezing of samples was avoided.

**Table 1:** Correlation analysis of s-GADD34-Ab levels using data from subjects in the CKD cohort.

	*rho* value	*p*-value
Age	0.0791	0.1220
Height	−0.0169	0.7409
Weight	0.0220	0.6668
BMI	0.0505	0.3234
Plaque score	0.1406	**0.0058**
Max-IMT	0.1370	**0.0076**
ABI (right)	−0.0490	0.3396
ABI (left)	−0.0917	0.0726
CAVI (right)	0.1371	**0.0077**
CAVI (left)	0.0941	0.0672
Dialysis Period	−0.0753	0.1410
HbA1c	−0.0338	0.6806
Whole parathyroid hormone	0.0595	0.2452
Kt/V	−0.0685	0.1807
Erythrocyte count	−0.0865	0.0904
White blood cell count	−0.0371	0.4685
Hemoglobin	−0.0723	0.1574
Hematocrit	−0.0731	0.1529
Platelet number	−0.1634	**0.0013**
Total protein	−0.0397	0.4374
Albumin	−0.0773	0.1307
Urea nitrogen	−0.0531	0.2994
Creatinin	−0.1029	**0.0439**
Uric acid	0.0032	0.9502
Ferritin	0.2112	**< 0.0001**
Fe	−0.0473	0.3552
Na	0.0156	0.7607
K	−0.0854	0.0945
Cl	−0.0021	0.9680
Ca	−0.0748	0.1435
P	0.0816	0.1102
Mg	0.0184	0.7193
AST	0.1250	**0.0142**
ALT	0.0942	0.0653
LDH	0.0500	0.3288
γ-GTP	0.0277	0.5891
Alkaline phosphatase	0.1028	**0.0440**
Total bilirubin	0.0457	0.3723
Amylase	−0.0660	0.1966
Creatine kinase	−0.0619	0.2265
Total cholesterol	−0.0537	0.2943
HDL cholesterol	−0.0759	0.1376
LDL cholesterol	−0.0739	0.1481
Triglyceride	0.0679	0.1842
CRP	0.0759	0.1377

Correlation coefficients (rho values) and *p*-values obtained by Spearman’s correlation analysis are shown. Demographic data include age, height, weight, BMI (body mass index), plaque score, maximum intima–media thickness (max-IMT), ankle brachial pressure index (ABI), cardio ankle vascular index (CAVI), dialysis duration, glycated hemoglobin (HbA1c), whole parathyroid hormone, standardized urea clearance (Kt/V), erythrocyte count, white blood cell count, hemoglobin, hematocrit, platelet number, total protein, albumin, urea nitrogen, creatinine, uric acid, ferritin, iron (Fe), sodium (Na), potassium (K), chlorine (Cl), calcium (Ca), inorganic phosphate (IP), magnesium (Mg), aspartate aminotransferase (AST), alanine amino transferase (ALT), lactate dehydrogenase (LDH), gamma-glutamyl transpeptidase (γ-GTP), alkaline phosphatase, total bilirubin, amylase, creatinine kinase, total cholesterol, high-density lipoprotein cholesterol (HDL cholesterol), low-density lipoprotein cholesterol (LDL cholesterol), triglyceride, and C-reactive protein (CRP). Significant correlations (*p* < 0.05) are shown in bold.

### 2.3. AlphaLISA (amplified luminescence proximity homogeneous assay-linked immunosorbent assay)

Antigenic GST-fused GADD34 was expressed in *Escherichia coli* and purified as described previously (12, 15). AlphaLISA was used to measure serum antibody levels. AlphaLISA was performed in 384-well microtiter plates (white opaque OptiPlate™ from Perkin Elmer, Waltham, MA) containing 2.5 μL of 100-fold diluted sera and 2.5 μL of GST-GADD34 or control GST protein (10 μg/mL) in AlphaLISA buffer (25 mM HEPES, pH 7.4, 0.1% casein, 0.5% Triton X-100, 1 mg/mL dextran-500, and 0.05% Proclin-300). The reaction mixture was incubated at room temperature for 6–8 h, followed by incubation with anti-human IgG-conjugated acceptor beads (2.5 μL at 40 μg/mL) and glutathione-conjugated donor beads (2.5 μL at 40 μg/mL) at room temperature in the dark for 1–14 days. Emissions were read on an EnSpire Alpha microplate reader (PerkinElmer) as described previously (15, 19–21). Specific reactions were calculated by subtracting the Alpha photon counts of the GST control from the counts of GST-fusion proteins.

### 2.4. Cells and culture conditions

The human osteosarcoma cell line, U2OS, human glioblastoma cell line, U87, and human lung carcinoma cell line, A549, were cultured in Dulbecco’s modified Eagle’s minimal essential medium supplemented with 10% (v/v) fetal bovine serum (Thermo Fisher Scientific, Waltham, MA) and 100 μg/mL of kanamycin (Meiji Seika, Tokyo, Japan) at 37°C in a humidified atmosphere containing 5% CO_2_.

### 2.5. Transfection of expression plasmids, shRNA, and siRNA

cDNA of GADD34 was obtained from GE Healthcare Life Sciences (Buckinghamshire, United Kingdom). The expression plasmids, pCMV-p53WT (22) and pCMV-MDM2, were provided by Dr. Bert Vogelstein (Howard Hughes Medical Institute). shRNA expression plasmids were purchased from Merck (MISSION^®^ shRNAs; Merck, Darmstadt, Germany). siRNA duplexes against GADD34, ATM, ATR, DNA-PK, and MDM2 as well as negative control scrambled siRNA were synthesized and purified by Merck. The sequences of siRNAs used in the present study were as follows:

GADD34 siRNA1: 5′-CUGUCUCCCAGCCUUCUGATT-3′;
GADD34 siRNA2: 5′-GAAAGGUGCGCUUCUCCGATT-3′;
GADD34 siRNA3: 5′-CUCUUCCACUCCUGCUACATT-3′;
ATM siRNA: 5′-CUUAGCAGGAGGUGUAAAUTT-3′;
MDM2 siRNA: 5′-CCACCUCACAGAUUCCAGCTT-3′.

Cells were transfected with expression plasmids and shRNA using FuGENE 6 and X-tremeGENE HP (Roche Diagnostics, Basel, Switzerland) or transfected with siRNA using Lipofectamine RNAiMAX (Thermo Fisher Scientific) according to the manufacturer’s instructions.

### 2.6. RT-PCR analysis

Total cellular RNA was extracted using RNeasy kits (QIAGEN). Relative mRNA expression levels were measured by RT-PCR using the SuperScript III One-Step RT-PCR System according to the manufacturer’s instructions (Cytiva, Pittsburgh, PA). Primer sequences used in the present study were as follows:

GADD34 forward, 5′-GGTCCTGGGAGTATCGTTCA-3′;
GADD34 reverse, 5′-CAGGGAGGACACTCAGCTTC-3′;
p53 forward, 5′-ACCTACCAGGGCAGCTACGGTTTC-3′;
p53 reverse, 5′-GCCGCCCATGCAGGAACTGTTACA-3′;
GAPDH forward, 5′-TCCACCACCCTGTTGCTGTA-3′;
GAPDH reverse, 5′-ACCACAGTCCATGCCATCAC-3′.

### 2.7. Luciferase assays

Firefly luciferase reporter plasmids, pG13-Luc (23) and PUMA-Luc (7), were provided by Dr. Bert Vogelstein (Howard Hughes Medical Institute). pGL3-p21-Luc (4) and pGL3-Bax-Luc (5) were provided by Dr. Mian Wu (University of Science and Technology of China). pGV-B2 Noxa-Luc (6) was provided by Dr. Nobuyuki Tanaka (Nippon Medical School). Decorin-Luc is a p53 reporter which contains the promoter region between positions −252 and −205 of the *decorine* gene (8). Cells seeded on 24-well plates were transfected with p53 expression plasmids together with firefly luciferase and a control *Renilla* luciferase reporter plasmid, SV40-Rluc, using FuGENE 6 and X-tremeGENE HP DNA Transfection Reagent (Roche Diagnostics). Two days after transfection, firefly and *Renilla* luciferase activities were determined using the Dual Luciferase Assay System (Promega) and a luminescencer (Atto, Tokyo, Japan) as previously reported (24). Firefly luciferase activities were normalized against *Renilla* luciferase activity.

### 2.8. Immunoprecipitation and Western blotting

Cells were suspended in 1% Nonidet P-40 lysis buffer [50 mM Tris–HCl (pH 7.6), 150 mM NaCl, 1% Nonidet P-40, 10 mM NaF, 1 mM Na_3_VO_3_, 1 mM dithiothreitol, 1 mM phenylmethylsulfonyl fluoride, 10 μg/mL leupeptin, and 10 μg/mL of pepstatin] and incubated on ice for 30 min. After centrifugation, cell lysates were immunoprecipitated with anti-GADD34 polyclonal antibody (Proteintech, Rosemont, IL) or Myc-Tag mouse monoclonal antibody (9B11, Roche Diagnostics) followed by precipitation using Protein G PLUS-Agarose Immunoprecipitation Reagent (Sepharose Bead Conjugate; Santa Cruz Biotechnology, Dallas, TX). Immunoprecipitates were washed three times with 0.1% Nonidet P-40 lysis buffer prior to use in Western blotting. Cell lysates or immunoprecipitated proteins were separated by SDS-polyacrylamide gel electrophoresis and transferred to nitrocellulose filters (Bio-Rad, Tokyo, Japan). Membranes were then incubated with anti-GADD34, anti-p53 (DO-1, Santa Cruz Biotechnology), anti-phospho-p53 (Ser-15) (Cell Signaling, Danvers, MA), anti-ATM (Santa Cruz Biotechnology), anti-MDM2 (SMP-14, Santa Cruz Biotechnology), anti-ubiquitin (Cell Signaling), anti-c-myc, anti-β-actin (Santa Cruz Biotechnology), or anti-α-tubulin (Merck) antibodies. Immunoreactive proteins were visualized using Immobilon Western Chemiluminescent Substrate (Merck).

### 2.9. Giemsa staining

Cells were fixed with 70% ethanol at room temperature for 30 min and stained with Giemsa solution (FUJIFILM Wako Pure Chemical Corporation).

## 3. Results

### 3.1. Serum anti-GADD34 antibody levels are higher in patients with AIS or CKD compared to HDs

**Figure 1:**
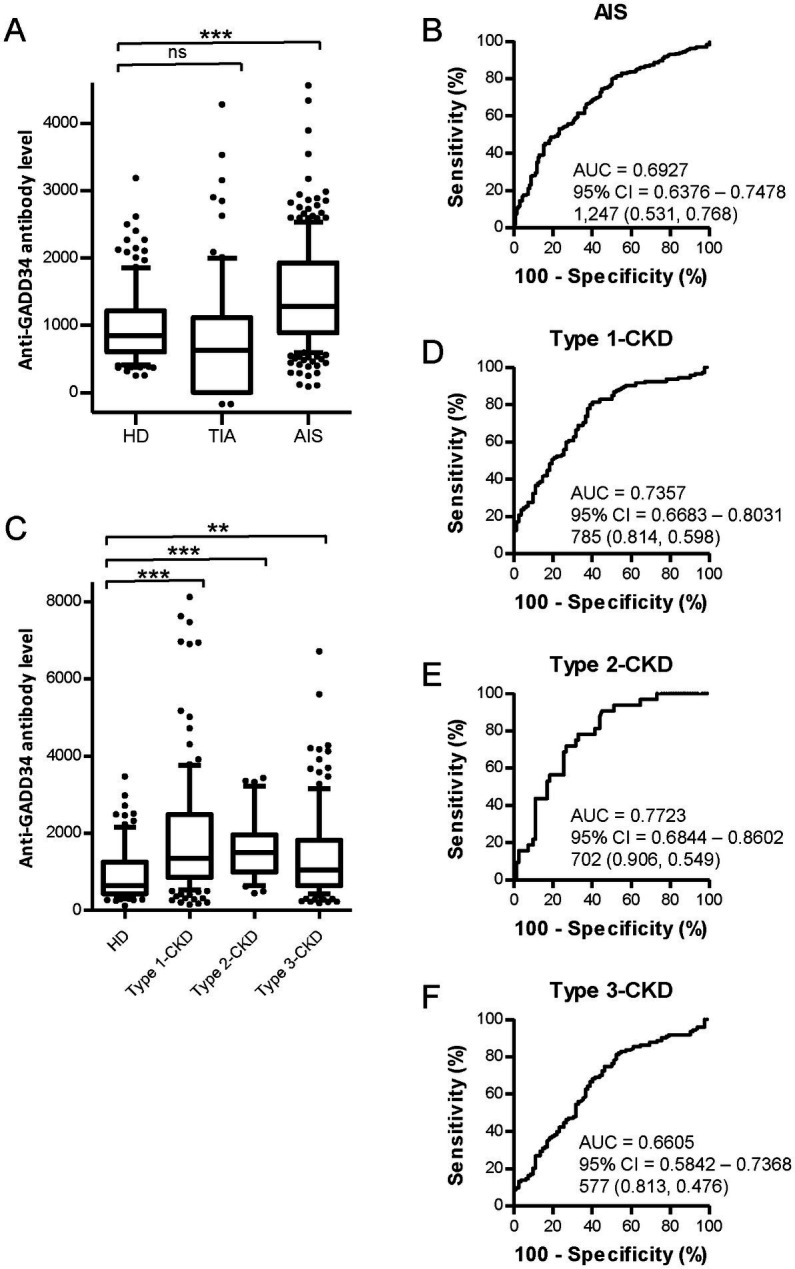
Comparison of serum GADD34 antibody levels between healthy donors (HDs) and patients with stroke or chronic kidney disease (CKD) by AlphaLISA. Serum samples from HDs and patients with AIS were obtained from Chiba Prefectural Sawara Hospital. Samples from patients with CKD were obtained from the Kumamoto cohort. Serum antibody levels in HDs and patients with transient ischemic attack (TIA) and acute ischemic stroke (AIS) **(A)** and patients with CKD **(C)** measured by AlphalLISA are shown by box-whisker plot. CKD types 1, 2, and 3 represent diabetic CKD, nephrosclerosis, and glomerulonephritis, respectively. Box plots display the 10th, 20th, 50th, 80th, and 90th percentiles. Total numbers of males and females, mean age ± standard deviation (SD), mean antibody levels ± SD, cutoff values, total numbers of individuals with anti-GADD34 antibodies, proportions (%) of individuals with anti-GADD34 antibodies, and *p*-values from HDs versus patients with TIA, AIS, and CKD are summarized and shown in Supplementary Tables S2, S3, respectively. *p*-values were calculated using the Kruskal-Wallis test. **, *p* < 0.01; ***, *p* < 0.001; ns, not significant. Receiver operating characteristic curve (ROC) analyses were performed to evaluate the utility of GADD34 antibodies in detecting AIS **(B)**, type-1 CKD **(D)**, type-2 CKD **(E)**, and type-3 CKD **(F)**. Values for area under the curve (AUC), 95% confidence intervals (CI), and cutoff values for serum anti-GADD34 antibody levels are shown. Sensitivity (left) and specificity (right) values are shown in parentheses.

GADD34 was identified by serological identification of antigens by recombinant cDNA expression cloning (SEREX) using sera of patients with atherosclerotic ischemic stroke and a human microvascular endothelial cell cDNA library (12). Serum anti-GADD34 antibody (s-GADD34-Ab) levels have previously been measured using ELISA; however, AlphaLISA can provide stable results with low background noise (15). s-GADD34-Ab levels were significantly higher in patients with AIS compared to HDs (*p* < 0.0001); however, no difference was observed between patients with TIA and HDs (Figure 1A). Mean (±SD) s-GADD34-Ab levels (Alpha counts) in HDs, patients with TIA, and patient with AIS were 992 (±552), 1,388 (±914), and 1,444 (±764), respectively. When a cutoff value representing the mean s-GADD34-Ab level in HDs plus two standard deviations was used (Alpha count = 2,095), the proportions of individuals with anti-GADD34 antibodies among HDs, patients with TIA, and patients with AIS were 6.5, 13.6, and 18.0%, respectively (Supplementary Table S2). Receiver operating curve (ROC) analyses were performed to evaluate the utility of s-GADD34-Ab levels in detecting AIS. The area under the curve (AUC) of GADD34-Abs for AIS was 0.6927 [95% confidence interval (CI), 0.6376–0.7478; Figure 1B]. When a cutoff value for s-GADD34-Ab levels of 1,247 determined according to the Youden index was used, the sensitivity and specificity of s-GADD34-Ab levels for the diagnosis of AIS were 53.1 and 76.8%, respectively.

We next measured s-GADD34-Ab levels in the sera of patients with CKD, which is closely associated with atherosclerosis. CKD was classified into three groups as follows: type 1, diabetic kidney disease; type 2, nephrosclerosis; and type 3, glomerulonephritis. Samples from patients with CKD were obtained from the Kumamoto cohort. Samples from HDs were obtained from Chiba University. All three CKD groups had significantly higher serum s-GADD34-Abs levels than HDs (Figure 1C). The proportions of individuals with detectable s-GADD34-Ab levels among HDs and patients with CKD types 1, 2, and 3 were 7.3, 27.6, 18.8, and 16.3%, respectively (Supplementary Table S3). ROC analyses demonstrated that AUC values for type-1, type-2, and type-3 CKD were 0.7357 (Figure 1D), 0.7723 (Figure 1E), and 0.6605 (Figure 1F), respectively.

We then performed Spearman correlation analyses to determine the correlation between s-GADD34-Ab levels and demographics in the Kumamoto cohort including age, sex, body height, weight, body mass index (BMI), degree of artery stenosis [plaque score, maximum intima–media thickness (max IMT), ankle brachial pressure index (ABI), and cardio ankle vascular index (CAVI)], dialysis duration, smoking, and serological parameters. Subject information was summarized in Supplementary Table S1. s-GADD34-Ab levels were significantly correlated with plaque score (*p* = 0.058), max IMT (*p* = 0.076), and CAVI (*p* = 0.077; Table 1), indicating that s-GADD34-Ab levels are associated with atherosclerosis. s-GADD34-Ab levels had the closest correlation with ferritin levels (*p* < 0.0001) but no correlation with serum iron concentrations or receipt of iron supplementation. s-GADD34-Ab levels also correlated with serum aspartate aminotransferase (*p* = 0.0142) and alkaline phosphatase (*p* = 0.044) levels and percutaneous transluminal angioplasty (PTA; *p* = 0.0023). Inverse correlations were observed between s-GADD34-Ab levels and serum platelet counts (*p* = 0.0013) and creatinine level (*p* = 0.0439). Serum HbA1c (glycated hemoglobin) levels, a surrogate of serum glucose levels, was not significantly correlated with s-GADD34-Ab levels.

### 3.2. Knockdown of GADD34 increases cell proliferation

**Figure 2:**
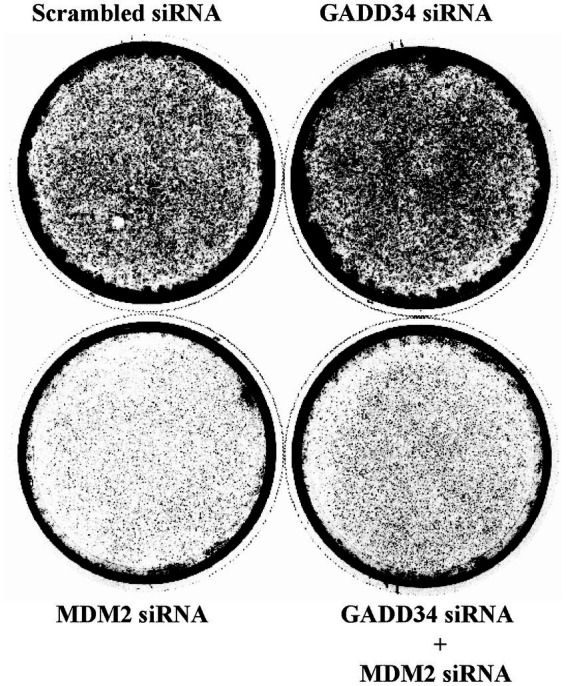
Knockdown of GADD34 increases cell proliferation. Human osteosarcoma U2OS cells were transfected with GADD34 siRNA and/or MDM2 siRNA. Scrambled siRNA was used as a control. Cells were cultured for 2 weeks, fixed, and stained with Giemsa.

GADD34 is known to be involved in cell proliferation (25). Endogenous GADD34 and MDM2 levels were down-regulated to investigate the effects of MDM2 and GADD34 on cell proliferation. Endogenous GADD34 and MDM2 were knocked down by transfection with GADD34 siRNA and/or MDM2 siRNA in the human osteosarcoma cell line, U2OS, followed by culture for 2 weeks. Cells fixed and stained with Giemsa demonstrated increased cell numbers after transfection with GADD34 siRNA compared to control siRNA (Figure 2). Conversely, transfection with MDM2 siRNA resulted in a marked reduction in the number of cells. Transfection with both GADD34 siRNA and MDM2 siRNA resulted in lower cell numbers compared to control siRNA or GADD siRNA, indicating that GADD34 siRNA increases proliferation while co-transfection with MDM2 and GADD34 siRNA markedly reduces proliferation. This suggests that GADD34 and MDM2 are interacted with each other in the regulation of cell proliferation.

### 3.3. Activation of p53 reporters by enforced expression of GADD34

**Figure 3:**
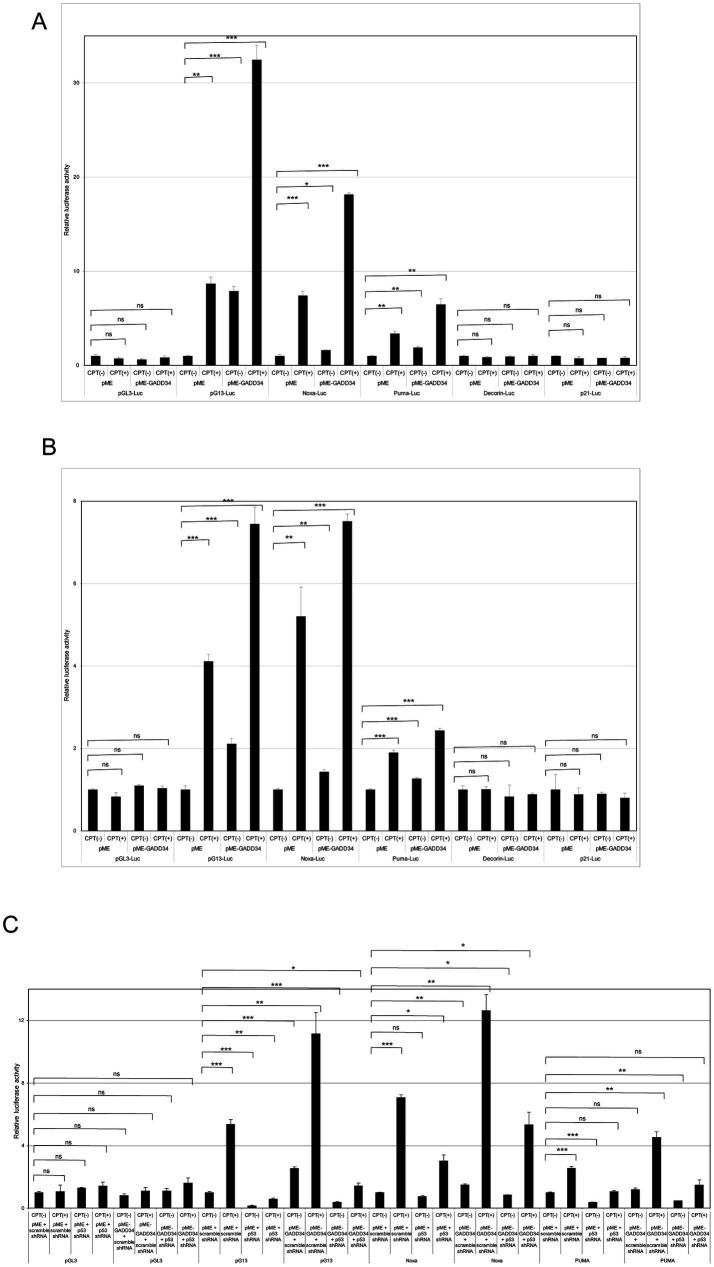
Activation of camptothecin (CPT)-activated p53 reporters by GADD34. **(A)** U2OS human osteosarcoma cells and **(B)** U87 human glioblastoma cells (5 × 10^4^ cells) were co-transfected with p53-responsive reporter plasmids (pG13-Luc, Noxa-Luc, PUMA-Luc, Decorin-Luc, and p21-Luc; 100 ng), transfection standard SV40-Rluc (10 ng), and the expression plasmid (500 ng) of pME-GADD34 or control empty vector pME-18S (pME). The control reporter plasmid, pGL3-Luc, was also used. The effects of knockdown of p53 were also examined by co-transfection with p53 shRNA or scrambled shRNA **(C)**. Cells were cultured for 24 h and then treated with CPT (1 μM) for 24 h. Relative firefly luciferase activities versus *Renilla* luciferase activities were measured in cell extracts. Error bars represent SD (*n* = 3). *, *p* < 0.05; **, *p* < 0.01; ***, *p* < 0.001; ns, not significant.

As p53 plays important roles in cell proliferation and apoptosis, the transactivation ability of p53 was examined using luciferase reporter assays. To achieve the enforced expression of GADD34, *GADD34* cDNA was inserted into the eukaryotic expression vector, pME-18S (designated as pME-GADD34), and a control empty vector, pME-18S (designated as pME). Transfection of pME-GADD34 induced the activation of p53-responsive reporters including pG13-Luc, Noxa-Luc, and PUMA-Luc but not Decorin-Luc or p21-Luc (Figure 3A). Treatment with a genotoxic anticancer drug, CPT, also activated these reporters, which was markedly enhanced in the presence of GADD34. On the other hand, CPT treatment has no effect on Decorin-Luc or p21-Luc.

A similar synergistic effect between GADD34 and CPT was observed in U87 glioblastoma cells (Figure 3B). When U2OS and U87 cells were treated with etoposide, an anticancer drug, pG13-Luc, Noxa-Luc and PUMA-Luc were activated and this effect was further augmented by the presence of GADD34 (Supplementary Figures S1A,B). Etoposide or GADD34 overexpression alone were unable to activate p21-Luc or the empty reporter, pGL3.

The effects of p53 activation in response to treatment with CPT or GADD34 overexpression was confirmed by knockdown of p53. After co-transfection of a p53 shRNA expression vector, a reporter plasmid, and a GADD34 expression plasmid, CPT treatment and/or GADD34 expression markedly attenuated the activation of pG13-Luc, Noxa-Luc, and PUMA-Luc (Figure 3C). No activation of luciferase reports was observed after transfection with scrambled shRNA as a control. Similar results were observed in response to treatment with etoposide (Supplementary Figure S1C). p53 shRNA alone reduced basal luciferase activity compared to scrambled shRNA, indicating that p53 retains some transcription activity in the absence of genotoxic stress.

### 3.4. p53 Protein levels are increased in the presence of GADD34

**Figure 4:**
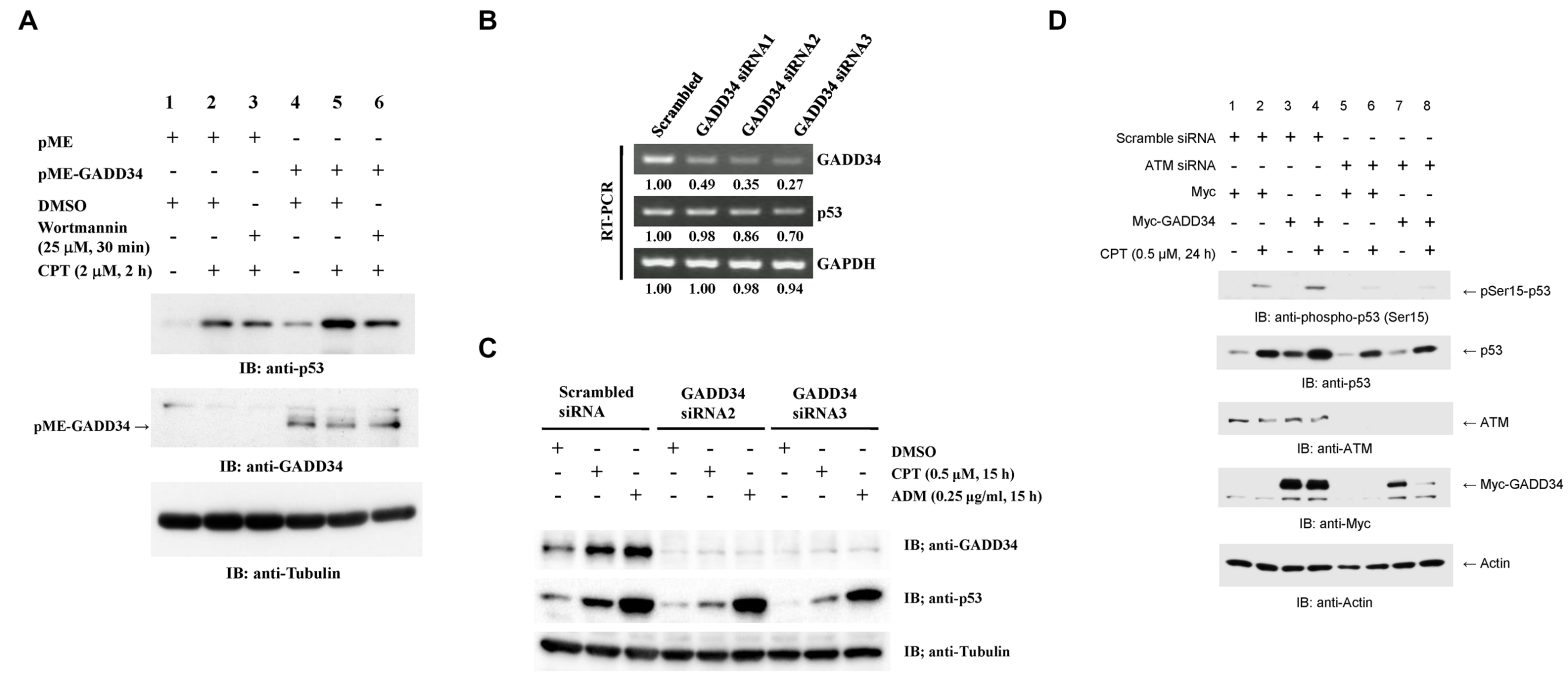
Increased p53 protein levels in the presence of GADD34. **(A)** Synergistic up-regulation of p53 protein by GADD34 and CPT. U2OS cells were transfected with pME-GADD34 or pME control vector and cultured for 48 h. Cells were pretreated with wortmannin (25 μM) or dimethyl sulfoxide (DMSO) for 30 min and then treated with CPT (2 μM) for 2 h. Total cell extracts were analyzed by Western blotting using anti-p53, anti-GADD34, and anti-α-tubulin antibodies. **(B)** Decreased GADD34 mRNA levels following treatment with siRNA. U2OS cells were transfected with three siRNAs including GADD34 siRNA1, siRNA2, and siRNA3, and cultured for 2 days. Then, total RNA was isolated and used as a template for reverse transcription followed by polymerase chain reaction (RT-PCR). Amplified DNA fragments of GADD34, p53, and control GAPDH are shown. Numbers indicate the relative amounts of DNA fragments versus cells transfected with scrambled siRNA. **(C)** Decreased GADD34 protein levels following treatment with siRNA. U2OS cells were transfected with GADD34 siRNA2, GADD34 siRNA3, and control scrambled siRNA and cultured for 33 h. Cells were then treated with CPT (0.5 μM), adriamycin (ADM; 0.25 μg/mL), or solvent DMSO (0.1%) for 15 h. Total cell extracts were analyzed by Western blotting using anti-GADD34, anti-p53, and anti-α-tubulin. **(D)** Increased p53 phosphorylation in the presence of GADD34. U2OS cells were transfected with ATM siRNA or control scrambled siRNA, cultured for 24 h, transfected with Myc-tagged GADD34 or control Myc-tagged expression vector (pcDNA-My), cultured for 24 h, then treated with CPT for further 24 h. Cells were harvested and subjected to Western blotting using anti-p53, anti-ATM, anti-Myc, and anti-β-actin antibodies.

The activation of p53 by GADD34 was further examined by Western blotting. p53 expression levels were almost undetectable after transfection with the control empty vector, pME. Increased p53 levels were observed after treatment with CPT (Figure 4A). ATM, ATR, and DNP-PK are well-known upstream activators of p53 (9). Increases in p53 levels were attenuated by pre-treatment with wortmannin, which inhibits ATM and DNA-PK. Enforced expression of GADD34 augmented p53 expression levels in non-treated, CPT-treated, and wortmannin plus CPT-treated cells. GADD34 levels were almost undetectable after transfection with pME but were clearly observed after transfection with pME-GADD34.

The effects of GADD34 on p53 expression were further examined by knockdown of GADD34. RT-PCR demonstrated GADD34 mRNA expression levels were reduced to <50% after transfection with siRNA-GADD34 (Figure 4B). GADD34 siRNA2 and GADD34 siRNA3 resulted in more potent knockdown of GADD34 mRNA levels than GADD34 siRNA1. This was further confirmed by Western blotting, with almost undetectable GADD34 protein levels in the presence of GADD34 siRNA2 or siRNA3 irrespective of treatment with CPT or ADM (Figure 4C). p53 expression levels in non-treated, CPT-treated, and ADM-treated cells were reduced in the presence of GADD34 siRNA. Thus, GADD34 promotes p53-mediated transcription by increasing p53 protein levels.

### 3.5. The activation of p53 by GADD34 is attenuated by co-transfection with ATM siRNA

The involvement of ATM in the upstream signaling pathway was further examined by Western blotting. p53 levels were synergistically increased by treatment with CPT and GADD34 overexpression (Figure 4D). Co-transfection with ATM siRNA reduced ATM protein levels to almost undetectable levels and decreased protein levels of p53 and GADD34. This finding implies that the protein levels of p53 and GADD34 may be regulated by an ATM-mediated pathway.

### 3.6. ATM functions upstream of GADD34 in response to DNA damage

**Figure 5:**
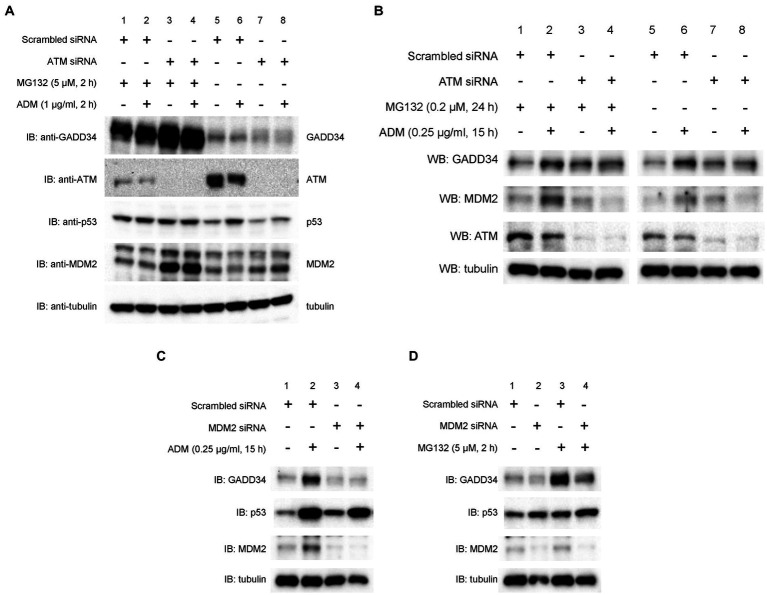
Suppression of ADM-induced increases in GADD34 protein levels by ATM siRNA and MDM2 siRNA. U2OS cells were transfected with **(A,B)** ATM siRNA, **(C,D)** MDM2 siRNA, or control scrambled siRNA and cultured for 24 h. **(A)** Cells were then treated with MG132 (5 μM) and/or ADM (1 μg/mL) for 2 h. **(B)** Cells were pretreated with MG132 (0.2 μM) for 9 h and then treated with ADM (0.25 μg/mL) for 15 h. Cells were treated with **(C)** ADM (0.25 μg/mL) for 15 h or **(D)** MG132 (5 μM) for 2 h. Total cell lysates were analyzed by Western blotting using anti-GADD34, anti-MDM2, anti-ATM, anti-p53, and anti-α-tubulin antibodies.

As p53 is rapidly degraded by the ubiquitin-proteasome system (26), the effects of a proteasome inhibitor, MG132, were examined. Surprisingly, knockdown of ATM markedly increased protein levels of GADD34 in the presence of MG132, whereas GADD34 protein levels were reduced in the absence of MG132 (Figure 5A). Protein levels of p53 and MDM2 increased after treatment with MG132. Thus, GADD34 protein levels may be regulated by the ubiquitin-proteasome system.

To investigate the effect of ATM on p53 activation, U2OS cells were transfected with ATM siRNA or scrambled siRNA and then treated with ADM. Western blotting demonstrated that ADM treatment increased protein levels of GADD34 and this effect was suppressed by knockdown of ATM (Figure 5B). The expression of ATM was efficiently reduced as expected. Pre-treatment with the proteasome inhibitor, MG132, partly increased protein levels of MDM2 and GADD34. Thus, ATM may function upstream of GADD34 and MDM2, possibly via proteasomal degradation.

### 3.7. MDM2 depletion results in GADD34 destabilization

To further investigate the effect of MDM2 on GADD34 expression, we measured protein levels using Western blotting. U2OS cells were transfected with scrambled siRNA or two different MDM2 siRNAs and then treated with the DNA-damaging agents, ADM or MG132. In control experiments, GADD34 levels increased after treatment with ADM or MG132. In contrast, MDM2 depletion attenuated the increase in GADD34 protein levels in response to ADM or MG132 (Figures 5C,D). These findings indicate that MDM2 expression results in GADD34 stabilization but not degradation.

### 3.8. Ubiquitination of GADD34 increases following treatment with genotoxic anticancer drugs

**Figure 6:**
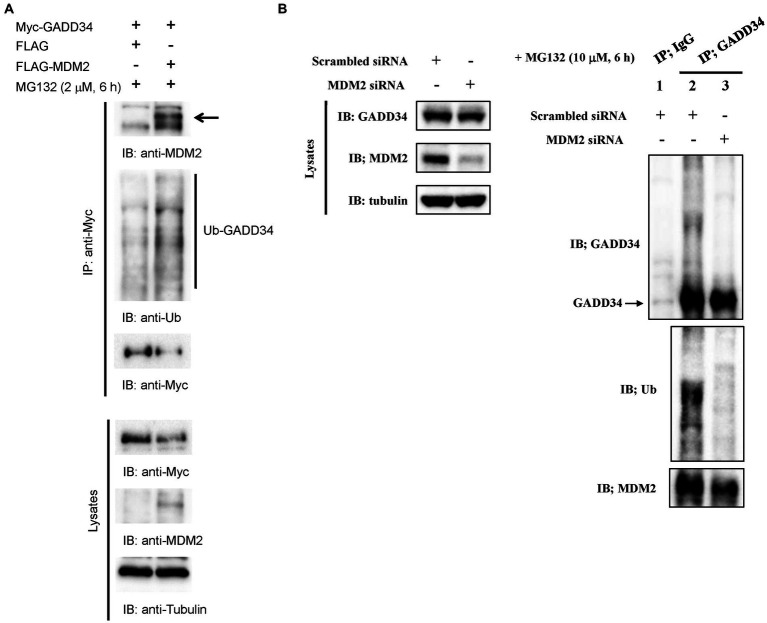
Ubiquitination of GADD34 via MDM2. **(A)** Increased ubiquitination of GADD34 following treatment with genotoxic anticancer drugs. U2OS cells were co-transfected with pcDNA-Myc-GADD34 and FLAG-MDM2 or the control FLAG vector and cultured for 48 h. Cells were then treated with MG132 (2 μM) for 6 h and then harvested. Cell extracts were immunoprecipitated using anti-Myc antibodies followed by Western blotting (IB) using anti-MDM2, anti-ubiquitin (Ub), and anti-Myc antibodies. Lysates without immunoprecipitation were analyzed using anti-Myc, anti-MDM2, and anti-α-tubulin antibodies. **(B)** Attenuation of GADD34 ubiquitination by MDM2 siRNA. A549 human lung carcinoma cells were transfected with MDM2 siRNA or control scrambled siRNA, cultured for 48 h, and then treated with MG132 (10 μM) for 6 h. Left panel, Western blotting of cell lysates using anti-GADD34, anti MDM2, and anti-α-tubulin antibodies. Right panel, cell lysates were immunoprecipitated with anti-GADD34 antibodies or control IgG and analyzed by Western blotting using anti-GADD34, anti-ubiquitin (Ub), anti-MDM2 antibodies.

We then examined ubiquitination of GADD34. Myc-tag GADD34 proteins in U2OS cell lysates were immunoprecipitated with anti-Myc antibody followed by Western blotting using anti-ubiquitin, anti-Myc, and anti-MDM2 antibodies. Ubiquitinated GADD34 was detectable in immunoprecipitates and was more clearly observed following treatment with MG132 (Figure 6A). Levels of ubiquitinated MDM2 were increased whereas levels of non-ubiquitinated GADD34 were decreased in response to MG132 treatment.

### 3.9. MDM2 depletion inhibited GADD34 ubiquitination

GADD34 has been shown to be ubiquitinated in cultured cells (27). To investigate the effect of MDM2 on GADD34 ubiqutination, U2OS cells were transfected with scrambled siRNA or MDM2 siRNA followed by treatment with MG132. GADD34 protein was immunoprecipitated with anti-GADD34 antibodies and then probed with anti-ubiquitin or anti-MDM2 antibodies. The detection of MDM2 in immunoprecipitates indicates binding between GADD34 and MDM2. Knockdown of MDM2 resulted in a marked reduction in GADD34 ubiquitination (Figure 6B). This finding indicates that the ubiquitination and expression levels of GADD34 are regulated by MDM2.

## 4. Discussion

GADD34 was discovered as a gene with increased expression in response to treatment with DNA-damaging agents (28). GADD34 was identified by SEREX screening using the sera of patients with atherosclerosis (12). In the present study, AlphaLISA analyses revealed that s-GADD34-Ab levels are significantly higher in patients with AIS or CKD compared to HDs (Figures 1A,C; Supplementary Tables S2, S3).

GADD34 siRNA increased cell proliferation, and this effects was attenuated by MDM2 siRNA (Figure 2). As the major ubiquitination target of MDM2 is p53 (10, 11), these findings indicate the inhibitory effect of GADD34 on cell proliferation may be partly mediated by the activity of p53. Luciferase reporter assays and Western blotting demonstrated increased protein levels of p53 in the presence of GADD34, indicating increased protein levels of p53 are responsible for the elevated transactivation ability of p53 after treatment with genotoxic anticancer drugs such as CPT, etoposide, and ADM (Figures 3, 4A,D; Supplementary Figures S1, S2). Increases in p53 protein levels in the presence of GADD34 were attenuated by treatment with wortmannin, an ATM inhibitor (Figure 4A). Similar attenuation of the increased p53 levels in the presence of GADD 34 was achieved by knockdown of ATM (Figure 4D). Further, Ser-15 phosphorylation of p53 may contribute to increased levels of p53 protein (Figure 4D).

p53 is known to be ubiquitinated by MDM2 leading to rapid degradation of p53 by proteasomes (29). GADD34 also appears to ubiquitinated by MDM2, with MDM2 siRNA found to reduce GADD34 ubiquitination (Figure 6B) and protein levels (Figure 5C) in the present study. That is, although GADD34 was ubiquitinated by MDM2, ubiquitinated GADD34 did not appear to be degraded. Continuous rather than transient binding of GADD34 to MDM2 may reduce degradation by proteasomes. These results indicate that GADD23 is ubiquitinated by MDM2 as an undegradable decoy which results in reduced ubiquitination and increased transactivation ability of p53.

Atherosclerosis is considered a disease of cell senescence. GADD34 reportedly induces p21 expression and cell senescence (30). Cell senescence via activation of p21 has also been shown to be induced by p53 (31). In the present study, p53 was activated by treatment with the genotoxic anticancer drugs, CPT, etoposide, and ADM (Figures 3, 4A,D; Supplementary Figures S1, S2), which induce DNA double-strand breaks (DSBs) (32–34). Similar DSBs had been shown to be induced by ischemia in rat brains (35). GADD34 protein levels increased after treatment with DSB-inducible CPT and ADM in the present study (Figure 4C), which may explain the elevated s-GADD34-Ab levels observed in patients with AIS (Figure 1A). Further, CPT and GADD34 synergistically activate p53 (Figures 3A,C) and increase p53 protein levels (Figure 4A) via ATM and MDM2, respectively.

Thus, ischemic stroke may activate p53 via DNA DSBs leading to neuronal injury mediated by autophagy and ferroptosis (36). Our correlation analysis of a CKD cohort demonstrated that s-GADD34-Ab levels are most closely correlated with serum ferritin levels (Table 1). Serum ferritin levels are a known risk factor for atherosclerotic heart failure (37). In addition, ferritin binds to p53 (38) and plays an essential role in ischemic stroke-induced hippocampal neuronal death via p53-mediated ferroptosis (39). As s-GADD34-Ab levels may be associated with neuronal cell death involving p53 and ferritin, serum anti-GADD34 antibody levels may have utility as a marker of neuronal ferroptosis.

## 5. Conclusion

p53 is activated by GADD34 via binding to MDM2 and this pathway may account for increased s-GADD34-Ab levels observed in patients with ischemic stroke. Serum anti-GADD34 antibodies may have utility as a marker of ischemia-induced neuronal injury.

## Data availability statement

The original contributions presented in the study are included in the article/Supplementary material, further inquiries can be directed to the corresponding authors.

## Ethics statement

The studies involving human participants were reviewed and approved by The Local Ethical Review Board of the Chiba University, Graduate School of Medicine in Chiba, Japan (approved nos. 2018-320 and 2020-1129). The patients/participants provided their written informed consent to participate in this study.

## Author contributions

YI, TH, and HK contributed to the conception and design of the study. NS, YY, SM, TM, and HK collected the research materials. GT, RN, NS, and KI performed the investigation. SM, TM, and YI performed the data curation and statistical analysis. GT, RN, and YY wrote the manuscript. TM, KI, and TH revised the manuscript. All authors contributed to the manuscript revision, read, and approved the submitted version.

## Funding

This work was partly supported by Grants-in-Aid of Japan Science and Technology Agency (exploratory research no. 14657335) and JSPS KAKINHI (grant numbers: 20K17953 and 19K09451).

## Conflict of interest

This work was performed in collaboration with Fujikura Kasei Co., Ltd. GT, RN, NS, and HK are employee of Fujikura Kasei Co., Ltd.

The remaining authors declare that the research was conducted in the absence of any commercial or financial relationships that could be construed as a potential conflict of interest.

## Publisher’s note

All claims expressed in this article are solely those of the authors and do not necessarily represent those of their affiliated organizations, or those of the publisher, the editors and the reviewers. Any product that may be evaluated in this article, or claim that may be made by its manufacturer, is not guaranteed or endorsed by the publisher.
