# Supplementary material for: GADD34 activates p53 and may have utility as a marker of atherosclerosis
Source: Front Med (Lausanne). 2023 May 9;10:1128921. doi: 10.3389/fmed.2023.1128921 (PMC10203227; doi:10.3389/fmed.2023.1128921)

Supplementary Material

GADD34 activates p53 and may have utility as a marker of atherosclerosis

**Go Tomiyoshi, Rika Nakamura, Natsuko Shinmen, Yoichi Yoshida, Seiichiro Mine, Toshio Machida, Katsuro Iwase, Yasuo Iwadate, Takaki Hiwasa* and Hideyuki Kuroda***

*** Correspondence:** Takaki Hiwasa, hiwasa_takaki@faculty.chiba-u.jp

Hideyuki Kuroda, h-kuroda@fkkasei.co.jpu

SUPPLEMENTARY TABLE S1

Subject information of Kumamoto CKD cohort

|  | Type-1 CKD | Type-2 CKD | Type-3 CKD |
| --- | --- | --- | --- |
| Total sample number | 145 | 32 | 123 |
| Sex (male/female) | 106/39 | 21/11 | 70/53 |
| Age | 66.0 ± 10.4 | 76.0 ± 9.8 | 62.0 ± 11.7 |
| Height (cm) | 161.1 ± 9.1 | 156.1 ± 9.8 | 159.5 ± 9.0 |
| Weight (kg) | 59.2 ± 12.2 | 53.9 ± 11.5 | 53.4 ± 11.2 |
| BMI (body mass index) | 22.8 ± 3.6 | 22.0 ± 3.1 | 20.8 ± 3.1 |
| Plaque score | 7.7 ± 4.1 | 6.8 ± 4.0 | 4.3 ± 3.6 |
| Maximum Intima media thickness (mm) | 2.2 ± 0.8 | 2.1 ± 0.7 | 1.8 ± 0.9 |
| ABI (ankle brachial index) (right) | 1.03 ± 0.18 | 1.04 ± 0.19 | 1.12 ± 0.14 |
| ABI (ankle brachial index) (left) | 1.03 ± 0.20 | 1.02 ± 0.20 | 1.11 ± 0.14 |
| CAVI (cardio-ankle vascular index) (right) | 9.8 ± 1.8 | 9.3 ± 2.3 | 9.0 ± 1.7 |
| CAVI (cardio-ankle vascular index) (left) | 9.8 ± 1.8 | 9.1 ± 2.5 | 8.8 ± 1.7 |
| Dialysis period (y) | 5.2 ± 3.9 | 4.9 ± 5.6 | 13.7 ± 10.0 |
| HbA1c (glycated hemoglobin) (%) | 6.0 ± 1.2 | 5.4 ± 0.8 | 5.5 ± 0.5 |
| Whole parathyroid hormone (pg/mL) | 69.3 ± 66.0 | 58.7 ± 54.9 | 70.3 ± 96.0 |
| Kt/V (standardized urea clearance) | 1.36 ± 0.23 | 1.33 ± 0.29 | 1.56 ± 0.25 |
| Red blood cell (×10^6^/μL) | 3.7 ± 0.5 | 3.5 ± 0.4 | 3.6 ± 0.4 |
| White blood cell (/μL) | 6013 ± 1825 | 5664 ± 1997 | 5415 ± 1416 |
| Hemoglobin (g\dL) | 10.7 ± 1.2 | 10.5 ± 1.0 | 10.7 ± 0.9 |
| Hematocrit (%) | 33.6 ± 3.7 | 33.4 ± 3.2 | 33.6 ± 2.9 |
| PLT (×10^3^/μL) | 164 ± 48 | 166 ± 48 | 169 ± 46 |
| Total protein (g/dL) | 6.7 ± 0.5 | 6.6 ± 0.4 | 6.6 ± 0.4 |
| Albumin (g/dL) | 3.8 ± 0.3 | 3.9 ± 0.3 | 3.9 ± 0.3 |
| Urea nitrogen (mg/dL) | 7.8 ± 1.3 | 8.0 ± 1.5 | 8.1 ± 1.1 |
| Creatinin (mg/dL) | 10.3 ± 2.4 | 9.8 ± 1.9 | 11.9 ± 2.3 |
| Uric acid (mg/dL) | 7.8 ± 1.3 | 8.0 ± 1.5 | 8.1 ± 1.1 |
| Ferritin (ng/mL) | 87.0 ± 110.0 | 87.2 ± 93.2 | 62.1 ± 89.0 |
| Fe (μg/dL) | 62.5 ± 28.0 | 63.4 ± 29.5 | 67.2 ± 23.1 |
| Na (mEq/L) | 137 ± 3 | 139 ± 2 | 139 ± 2 |
| K (mq/L) | 4.7 ± 0.8 | 4.6 ± 0.6 | 4.9 ± 0.6 |
| Cl (mEq/L) | 102 ± 3 | 103 ± 3 | 102 ± 3 |
| Ca (mEq/L) | 9.1 ± 0.7 | 9.5 ± 0.9 | 9.3 ± 0.7 |
| Inorganic phosphate (mEq/L) | 5.6 ± 1.3 | 5.2 ± 1.3 | 5.4 ± 1.1 |
| Mg (mEq/L) | 2.7 ± 0.4 | 2.7 ± 0.4 | 2.8 ± 0.4 |
| Aspartate aminotransferase (U/L) | 12.9 ± 6.2 | 13.9 ± 5.1 | 14.4 ± 8.0 |
| Alanine aminotransferase (U/L) | 11.7 ± 6.7 | 10.1 ± 3.8 | 11.5 ± 7.1 |
| Lactate dehydrogenase (U/L) | 185 ± 36 | 197 ± 39 | 191 ± 33 |
| γ-Glutamyl transpeptidase (U/L) | 32.4 ± 49.6 | 17.8 ± 6.4 | 29.4 ± 30.4 |
| Alkaline phosphatase (U/L) | 244 ± 102 | 206 ± 63 | 242 ± 109 |
| Total bilirubin (mg/dL) | 0.4 ± 0.1 | 0.4 ± 0.1 | 0.3 ± 0.1 |
| Amylase (U/L) | 103 ± 40 | 123 ± 50 | 143 ± 56 |
| Creatine kinase (U/L) | 98 ± 63 | 97 ± 70 | 136 ± 172 |
| Total cholesterol (mg/dL) | 146 ± 30 | 152 ± 20 | 163 ± 33 |
| HDL-cholesterol (mg/dL) | 44.8 ± 14.3 | 41.4 ± 11.3 | 51.7 ± 14.6 |
| LDL-cholesterol (mg/dL) | 73.8 ± 22.5 | 81.8 ± 15.2 | 85.0 ± 26.6 |
| Triglyceride (mg/dL) | 114 ± 85 | 112 ± 61 | 107 ± 69 |
| C-reactive protein (mg/dL) | 2.5 ± 5.4 | 2.6 ± 6.2 | 1.4 ± 2.1 |

Subject information of Kumamoto chronic kidney disease (CKD) cohort was summarized including age, sex (numbers of male and female), and blood test data (average ± SD).

SUPPLEMENTARY TABLE S2

Comparison of the serum antibody levels of healthy donors (HDs) vs. those of patients with transient ischemic attack (TIA) or acute ischemic stroke (AIS).

| Sample information | | HD | TIA | AIS |
| --- | --- | --- | --- | --- |
|  | Total sample number | 139 | 44 | 228 |
|  | Male/Female | 87/52 | 23/21 | 130/98 |
|  | Age (Average ± SD) | 51.8 ± 12.7 | 68.5 ± 12.1 | 77.0 ± 11.1 |
|  |  |  |  |  |
| Alpha analysis (antibody level) | | s-GADD34-Ab |  |  |
| HD | Average | 992 |  |  |
|  | SD | 552 |  |  |
|  | Cutoff value | 2,095 |  |  |
|  | Positive No. | 9 |  |  |
|  | Positive (%) | 6.5% |  |  |
| TIA | Average | 1,388 |  |  |
|  | SD | 914 |  |  |
|  | Positive No. | 6 |  |  |
|  | Positive (%) | **13.6%** |  |  |
|  | *P* (TIA vs HD) | ns |  |  |
| AIS | Average | 1,444 |  |  |
|  | SD | 764 |  |  |
|  | Positive No. | 41 |  |  |
|  | Positive (%) | **18.0%** |  |  |
|  | *P* (AIS vs HD) | **<0.001** |  |  |

Upper panel indicates the numbers of total samples and samples from male and female samples as well as ages [average ± standard deviation (SD)]. The lower panel summarizes the serum antibody levels (Alpha photon counts) examined by AlphaLISA using purified GADD34-GST protein as an antigen. Cutoff values were determined as the average HD values plus two SD, and positive samples higher than the cutoff value were scored. *P* values were calculated using the Kruskal-Wallis test. *P* values <0.05 and positive rates >10% are marked in bold text. A scatter dot plot of the same results is shown in Figure 1A.

SUPPLEMENTARY TABLE S3

Comparison of s-GADD34-Ab levels of HDs versus those of patients with chronic kidney disease (CKD).

| Sample information | | HD | Type-1 CKD | Type-2 CKD | Type-3 CKD |
| --- | --- | --- | --- | --- | --- |
|  | Total sample number | 82 | 145 | 32 | 123 |
|  | Male/Female | 44/38 | 106/39 | 21/11 | 70/53 |
|  | Age (Average ± SD) | 44.1 ± 11.2 | 66.0 ± 10.4 | 76.0 ± 9.8 | 62.0 ± 11.7 |
|  |  |  |  |  |  |
| Alpha analysis (antibody level) | | s-GADD34-Ab |  |  |  |
| HD | Average | 923 |  |  |  |
|  | SD | 723 |  |  |  |
|  | Cutoff value | 2,369 |  |  |  |
|  | Positive No. | 6 |  |  |  |
|  | Positive rate (%) | 7.3% |  |  |  |
| Type-1 CKD | Average | 2,006 |  |  |  |
|  | SD | 2,085 |  |  |  |
|  | Positive No. | 40 |  |  |  |
|  | Positive rate (%) | **27.6%** |  |  |  |
|  | *P* (vs HD) | **<0.001** |  |  |  |
| Type-2 CKD | Average | 1,635 |  |  |  |
|  | SD | 850 |  |  |  |
|  | Positive No. | 6 |  |  |  |
|  | Positive rate (%) | **18.8%** |  |  |  |
|  | *P* (vs HD) | **<0.001** |  |  |  |
| Type-3 CKD | Average | 1,438 |  |  |  |
|  | SD | 1,158 |  |  |  |
|  | Positive No. | 20 |  |  |  |
|  | Positive rate (%) | **16.3%** |  |  |  |
|  | *P* (vs HD) | **<0.01** |  |  |  |

Types-1, -2, and -3 CKDs correspond to diabetic kidney disease, nephrosclerosis, and glomerulonephritis, respectively. The upper panel indicates the numbers of all samples and samples from males and females as well as age (average ± SD). The lower panel summarizes the serum antibody levels examined by AlphaLISA using purified GADD34-GST protein as an antigen as described in the legend of Table S1. *P* values <0.05 and positive rates >10% are marked in bold text. A scatter dot plot of the same results is shown in Figure 1C.

SUPPLEMENTARY FIGURE S1

Stimulation of etoposide (Etop)-activated p53 reporters by GADD34. U2OS human osteosarcoma cells (A, C) and U87 human glioblastoma cells (B) (5 × 10^4^ cells) were co-transfected with p53-responsive reporter plasmids (pG13-Luc, Noxa-Luc, Puma-Luc, and p21-Luc; 100 ng), transfection standard SV40-Rluc (10 ng), and the expression plasmid (500 ng) of pME-GADD34 or control empty vector pME-18S. Control reporter plasmid, pGL3-Luc was also used. p53 shRNA and control scrambled shRNA were also co-transfected in C. Cells were cultured for 48 h and then treated with etoposide (Etop, 5 µM) for 24 h. Luciferase activities in the cell extracts were measured. The error bars represent S.D. (n = 3). * P <0.05, ** P <0.01, *** P <0.001, ns: not significant.


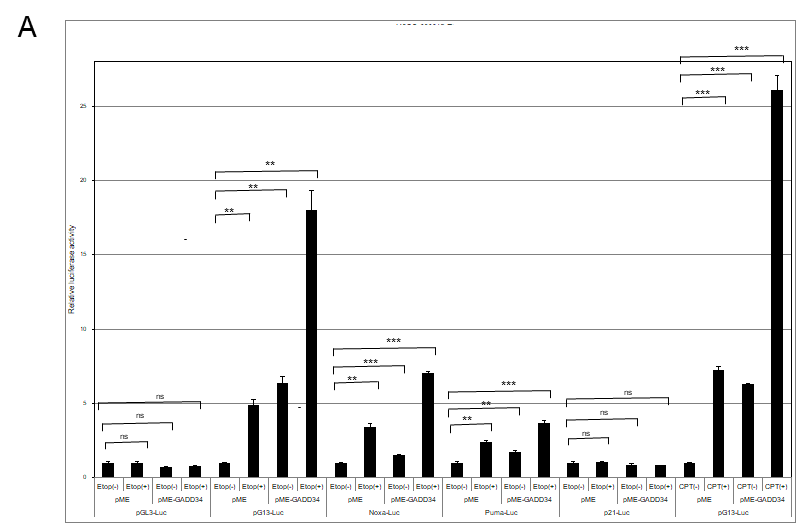


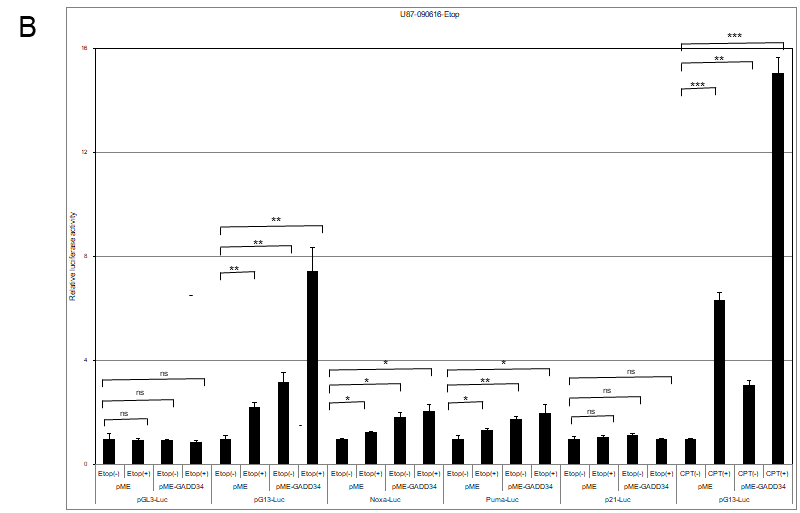


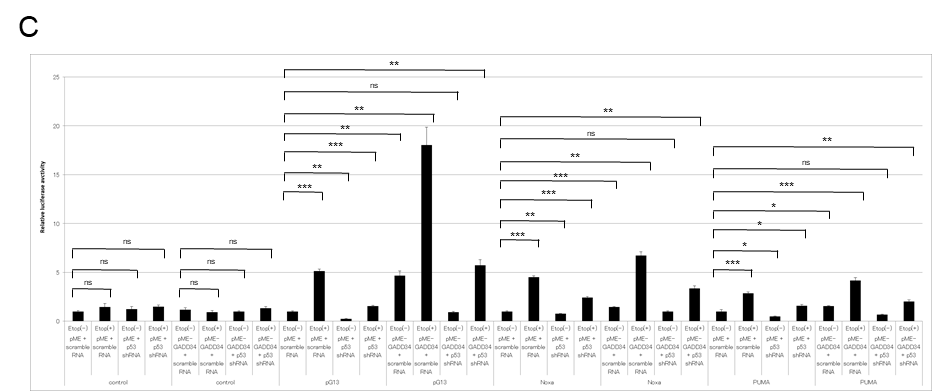

Supplement: Supplementary file 1 [file Table_1.DOCX]
